# New Method of Forming an Inlay Matrix

**Published:** 1904-05-15

**Authors:** Charles Channing Allen


### NEW METHOD OF FORMING AN INLAY MATRIX.

BY CHARLES CHANNING ALLEN, D.D.S.

In making a gold or platinum foil matrix, great difficulty is often experienced in removing the matrix from the cavity without distortion. Often if an approximal matrix has been formed, it requires the greatest care and most skillful manipulation to get it from the mouth into an investment without some change of shape. It has been suggested that, in order to facilitate the forming and removing of a matrix, it be previously filled with some material solid enough that it may be handled.

Many substances have been tried for this, but the difficulty has always been to remove the material without in some way damaging the matrix. Some of these materials can be removed by melting, burning, washing, etc., but at the expense of a good deal of time and annoyance. Wax or gutta-percha leaves an ash or deposit or some trace of their occupancy behind them. This is of course undesirable, as the minutest trace of alien matter will frequently ruin the finished inlay by causing discoloration, bubbles, etc. After trying all of the materials heretofore used that have been brought to my notice, I began experimenting with gum camphor, the ordinary article procurable at the drug store. I find that this can be packed into the matrix and greatly facilitates forming it, as it has the desirable flow and packs very hard, thus actually swaging the matrix to all cavity walls and enabling you to burnish down the edges until they are smooth and perfectly adapted to the tooth.

One can finish and rub the edges of the matrix of either gold or platinum foil, until he is satisfied that the fit is perfect, without any fear of drawing away at any point.

Very little manipulation with spunk, cotton or bibulous paper is necessary before resorting to camphor. A small lump of camphor can be placed in the cavity and burnished down with a hard pressure, and the result will be surprisingly gratifying. If the matrix is difficult of removal, sharp instruments, such as an explorer, may be inserted in the body of the camphor, and the matrix, camphor and all, with a little care, be removed from a location where it would be impossible to get a matrix out without distortion unless it was filled. The camphor, when properly placed, comes out hard enough to stand all the handling necessary. After the matrix is removed, you can proceed to invest in asbestos or any compound used for that purpose without fear of disturbing the shape of your matrix. But now comes the best part of the whole scheme; if you are investing, using alcohol, all you have to do is to touch a match to the investment, and by the time the alcohol is burned up, the camphor will have absolutely disappeared, leaving a perfectly clean matrix with no trace of ash or any residue remaining. This is of course the very thing we want. You now go ahead with your work with the assurance that it will be clean. Alcohol is not necessary to the removal of the camphor, as it will burn up without its aid if you should invest with a mixture using water.

In case the camphor has been somewhat contaminated with blood from the gum in making, the whole body of the camphor may be removed, blood and all, after soaking a few moments in alcohol. In such a case, that procedure is better than burning.

With this method pins or staples may be used in your matrix and the camphor packed hard enough around them so that they may be removed and invested at once without any fear of displacement. In fact, camphor lends itself almost perfectly to many uses in inlay and porcelain work. Pins may be held in position in making the platinum base for molar crowns in this manner, etc. The camphor has no unpleasant qualities to interfere with using it in the mouth.

A little experience will enable one to fill out approximate contours with the camphor, which, even after they have been removed, will prove serviceable in guiding the eye to contouring work.—*Western Dental Journal.*

